# Hepatitis B virus and inhibitor of apoptosis proteins – a vulnerable liaison

**DOI:** 10.1038/cddiscovery.2016.14

**Published:** 2016-02-22

**Authors:** G Ebert, M Pellegrini

**Affiliations:** 1 Division of Infection and Immunity, The Walter and Eliza Hall Institute of Medical Research, Parkville, Victoria , Australia; 2 Department of Medical Biology, The University of Melbourne, Parkville, Victoria , Australia

## Hepatitis B infection and current therapies

The treatment for hepatitis C virus (HCV) infection has been revolutionized by the introduction of highly potent antiviral drugs that can successfully cure most patients.^1^ A major distinguishing feature between HCV and HBV infection is that the latter forms a persistent mini-chromosome and it integrates into the host genome, whereas HCV is localised solely to the cytoplasmic compartment. Our current HBV antiviral drugs are highly effective in suppressing viral replication, but they cannot cure the infection that is present in >2 billion people worldwide.^2^ The immune system of most of these people is capable of effectively controlling the acute phase of HBV infection. However, a proportion of people with HBV infection have persistent viral replication and chronic inflammation that predisposes them to cirrhosis and hepatocellular carcinoma (HCC).

A vaccine has been available since the early 1980s that is highly effective in preventing HBV infection, but it has no therapeutic efficacy for the 360 million people who are chronically infected with HBV and have persistent disease.^2^ The replicative episomal form of HBV DNA, called covalently closed circular (ccc) DNA, prevents current therapies, including antiviral drugs, from being curative. These drugs need to be taken indefinitely to prevent viral relapse.^3^

Strengthened by the advent of HCV curative treatments, there is a huge interest in developing curative therapies for HBV infection. Potential new therapies for chronic hepatitis B include direct-acting antivirals, such as viral assembly inhibitors, gene silencing approaches and viral entry inhibitors.^3^ All of these therapies primarily target the virus itself and indeed many, if not most of our anti-infective agents rely on interfering with microbial proteins or the microbial genome to interrupt the ability of the pathogens to replicate.

A less explored avenue for the treatment of chronic infections, which may offer enormous potential, is targeting host cell factors that modulate cell signalling, innate or adaptive immune responses. Interferon therapy is an established method of modulating host responses to HBV infection and toll-like receptor agonists (e.g., TLR7 agonist) are currently being tested.^3^ However, to date none of these treatments have shown great efficacy in curing HBV infection.

## TNF signalling and HBV infection

Recently, we developed a completely novel approach of inducing death of HBV-infected hepatocytes to eliminate the viral reservoir and cure infection in a small animal model.^4,5^ If this success translates to efficacy in clinical trials, infection can be eliminated along with the nidus for HCC development. Such a therapeutic intervention to combat chronic HBV infection has not been explored before. We discovered that gene-targeted mice lacking specific cellular IAPs (c-IAP1 and c-IAP2) were able to quickly and efficiently eliminate HBV-infected cells without causing overt collateral damage.^4^

IAPs are central critical regulators of a large number of cell-signalling pathways involved in the immune response, but also in regulating survival and cell death signalling downstream of death receptors. Our discoveries, potentially, had immediate therapeutic implications because IAP antagonists were already in clinical trials for the treatment of cancers. Birinapant is an example of an IAP antagonist currently being investigated in cancer clinical trials. We found that it effectively antagonised IAPs in hepatocytes and it promoted TNF-dependent elimination of HBV and cured infection in preclinical models.^5^

HBV is considered a non-cytopathic virus and it may utilise diverse mechanisms to abrogate TNF-mediated antiviral responses to infection. Indeed, TNF siganling is hijacked by HBV to enhance NF-κB transcriptional activity, and promote cell survival and activation to facilitate viral replication.^6,7^ In contrast, other studies have suggested that TNF may abrogate HBV replication by deregulating hepatocyte nuclear factors.^8^ Recent work implicated a role for TNF and IFNgamma, produced by T cells, in promoting non-cytolytic control of chronic HBV infection by diminishing the pool of cccDNA.^9^

TNF-mediated cell survival signalling is tightly regulated by IAPs, which function as ubiquitin E3 ligases via their RING domain.^10^ We observed no marked changes in c-IAP1 and XIAP levels in the liver during the first weeks after induction of HBV infection in our animal studies.^4^ Assessment of mouse c-IAP2 protein levels was not possible in these studies as there was no reliable antibody against this protein. Regardless of whether the levels of IAPs change during HBV infection, we found that c-IAP1 and c-IAP2 prevent the clearance of HBV infection.^4^ The therapeutic implications of this discovery were immediately tangible because IAP antagonists were already in clinical trials for other indications.

These drugs mimic the activity of an endogenous inhibitor of IAP function called Smac/Diablo. The small-molecule compounds called Smac mimetics were designed to mimic the inhibition of IAPs, antagonise their function and induce TNF-dependent cell death.^11^ Multiple clinical cancer trials validated the therapeutic applicability of Smac mimetics to induce targeted TNF-mediated death of tumour cells.

## HBV and IAPs – a vulnerable liaison

In a completely novel approach, we used the Smac mimetic birinapant to reroute the signalling activity of endogenous TNF away from NF-κB activation and towards cell death induction in HBV-infected hepatocytes. We exploited the vulnerability created by HBV – a dependence on TNF/NF-κB and we harnessed the activity of endogenous TNF to kill cells in the absence IAPs. In our immunocompetent mouse model of chronic HBV infection, the Smac mimetic birinapant promoted TNF-mediated apoptosis of infected hepatocytes. Moreover, our study showed that birinapant preferentially killed infected cells over uninfected cells due to the vulnerability created by the virus.^5^ In addition, the ability of the immune system to localise production of TNF at the site of infection may mitigate collateral damage.

Our Smac mimetic therapeutic approach takes advantage of an Achilles heel in HBV’s attempt to utilise TNF to promote host cell survival and NF-κB activity. HBV X protein was shown to upregulate TNF expression.^4,6^ TNF and other pro-inflammatory cytokines in turn increase stability of the HBV X protein and they increase NF-κB signalling, which is essential for HBV replication.^7^ But the molecular details and mechanism of how HBV replication affects cell signalling and regulates induction of apoptosis or necroptosis warrant further investigations.

Another virus that has hijacked NF-κB signalling is HIV. In this case too much or too little NF-κB activity can cause problems for the host. HIV requires NF-κB for viral replication, but in the absence of sufficient. NF-κB activity HIV can adopt a quiescent latent integrated form. Latently infected cells are a dormant viral reservoir of HIV that can reseed the viral pool as soon as conventional antiviral therapy is stopped, thus preventing HIV cure. If all latently infected cells could be reactivated and killed, then the viral reservoir could be eliminated. It was recently shown that Smac mimetic-mediated depletion of c-IAP1, which is also a repressor of non-canonical NF-κB signalling, could activate transcriptional activity and reverse HIV-1 latency.^12^ We would speculate that additionally Smac mimetics could promote killing of the reactivated HIV-infected cells.

Our work demonstrated that we are able to beat the HBV virus at its own game. In a completely novel approach, we were able to promote TNF-mediated apoptosis of HBV-infected cells by taking advantage of the viruses addiction to TNF.^5^
